# Public health round-up

**DOI:** 10.2471/BLT.20.011020

**Published:** 2020-10-01

**Polio progress**

An infant receives polio vaccine at the Jupanziri Health Centre III in Nebbi district, Uganda. The country hasn't reported a wild polio case since November 2010. The WHO African Region was declared free of wild poliovirus on 25 August 2020. However, vaccine-derived poliovirus is circulating in 16 countries in the African Region.

**Figure:**
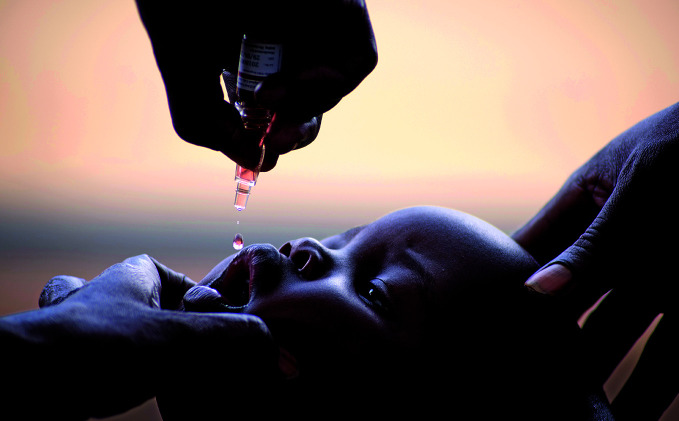
UNICEF/Henry Bongyereirwe

## Global vaccine initiative funding crunch

There is an urgent need for US$ 15 billion for the Access to COVID-19 Tools (ACT) Accelerator, a global initiative launched to boost the development, production and equitable distribution of COVID-19 tests, treatments and vaccines.

A call for funding was made by the United Nations Secretary-General, António Guterres, at the inaugural meeting of the Accelerator Facilitation Council, which took place on 10 September.

According to the Secretary-General, a total of US$ 35 billion is still needed for the ACT-Accelerator to realise its goals of producing 2 billion vaccine doses, 245 million treatments and 500 million tests by 2021.

“There is a real urgency in these numbers,” said Secretary-General Guterres. “Without an infusion of US$ 15 billion over the next three months, beginning immediately, we will lose the window of opportunity.”

The ACT-Accelerator increases the chance of positive outcomes for all countries by sharing the costs and benefits of research and development and facilitating accelerated access to needed medical products.

https://bit.ly/2RqKsGT

## Africa wild polio free

The WHO African Region was certified free of wild poliovirus. The Africa Regional Certification Commission issued the certification on 25 August after four years without a reported case of polio due to wild poliovirus in the region. Five of the six WHO regions – home to over 90% of the world’s population – are now free of the wild poliovirus.

While a significant milestone, it is vital that efforts to support and strengthen immunization programmes and health systems in the African Region are maintained to hold ground against wild polio and to tackle the spread of type 2 circulating vaccine-derived poliovirus (cVDPV2), which is present in 16 African countries. Pockets of low population immunity continue to expose people to the risk of polio caused by cVDPV2 infection, a situation exacerbated by interruptions in vaccination due to COVID-19.

Pakistan and Afghanistan are the only two countries that continue to report cases of infection with wild poliovirus.

https://bit.ly/2FAvYBp

## Vaccine-derived poliovirus detected in Sudan

The Federal Ministry of Health, Sudan notified WHO of the detection of cVDPV2 in the country and reported a total of 13 cVDPV2 infections between 9 August and 26 August. According to a notification on 9 August, the virus is genetically linked to those causing infections in neighbouring Chad.

On 1 September WHO assessed the risk of further international spread of cVDPV2 across central Africa and the Horn of Africa to be high.

https://bit.ly/2ZAYPNi

## Pandemic response failure

A new report by the Global Preparedness Monitoring Board (GPMB) offers a harsh assessment of the global COVID-19 response, calling it “a collective failure to take pandemic prevention, preparedness, and response seriously and prioritize it accordingly.”

Published on 14 September, the GPMB’s second report highlights the lack of multilateral cooperation in responding to the ongoing COVID-19 pandemic, citing “geopolitical tensions” as an obstacle to leadership by the G7, G20 and multilateral organizations. The report calls on leaders to renew their commitment to the multilateral system and strengthen WHO as an impartial and independent international organization.

“Viruses don’t respect borders. The only way out of this devastating pandemic is along the path of collective action, which demands a strong and effective multilateral system,” said Dr Gro Harlem Brundtland, co-chair of the GPMB, and former WHO Director-General.

The GPMB was created in response to recommendations of the UN Secretary-General’s Global Health Crises Task Force in 2017 and is co-convened by WHO and the World Bank Group.

https://bit.ly/2E5hhpI

## Under-five mortality falls

Under-five mortality rates declined by almost 60% between 1990 and 2019, dropping from an estimated 93 to 38 deaths per 1000 children (12.5 million to 5.2 million). This is according to the latest *Levels and trends in child mortality: report 2020,* released by the United Nations Children’s Fund (UNICEF) and partners on 9 September.

The report presents a comprehensive range of child mortality rates across the world and for the first time includes estimates for youth aged 15–24 years – as well as the progress made toward meeting the 2030 sustainable development goal targets.

Despite progress, the report flags concern over the likely impact of the ongoing novel coronavirus disease (COVID-19) pandemic, and states that for mortality targets to be met, the rate of progress needs to accelerate.

According to the report, an estimated 7.4 million children and young people under the age of 25 died in 2019 largely due to treatable causes such as infectious diseases. More than 5 million children died before reaching age 5, and nearly half of those deaths were among newborns.

https://bit.ly/3mh3yNP

## Pandemic hits services

Around nine in ten countries experienced disruptions to health services between March and June. This is according to the *Pulse survey on continuity of essential health services during the COVID-19 pandemic*, a first indicative survey on the impact of COVID-19 on health systems which was published by WHO on 31 August.

Based on reports from senior health ministry officials from 105 countries across the income spectrum, the survey indicates that low- and middle-income countries (LMIC) experienced the greatest difficulties, that most countries reported disruptions to routine and elective services, while critical care – such as cancer screening and treatment and HIV therapy – was significantly disrupted in low-income countries.

"The survey shines a light on the cracks in our health systems, but it also serves to inform new strategies to improve healthcare provision during the pandemic and beyond,” said Tedros Adhanom Ghebreyesus, WHO Director-General.

https://bit.ly/3kfBt7C

## Under-resourced Ebola response

WHO is having to use emergency funds to support epidemiological and public health interventions in the Équateur Province of the Democratic Republic of the Congo, where an outbreak of Ebola virus disease continues to spread. As of 3 September, 110 people had been reported to be infected with the virus, 47 of whom had died.

The response is encountering multiple obstacles, including community resistance to blood sampling and recommended safe burial practices, and strikes among locally-based response teams.

WHO is working with the Ministry of Health and partners to address these challenges, but a lack of funding from partners since the start of 2020 is hampering their efforts. Access to financial and human resources are further challenged by the ongoing COVID-19 outbreak which is putting an additional burden on the national health system.

https://bit.ly/3ixEr6X

## New WHO expert group

The World Health Organization convened its first technical advisory group on behavioural insights and sciences for health. Comprised of experts from a range of disciplines, including anthropology, psychology and social marketing, the group’s formation was announced on 3 September as part of a new initiative designed to tap into social and behavioural sciences to inform policy formulation and implementation.

The group will also support WHO’s regional and country offices in gathering local, context-specific evidence for a better understanding of how individual and community behaviour influences health.

https://bit.ly/3c32rwy

**Cover photo**

A mother brings her daughter for postnatal care at the Greater Accra Regional Hospital in Ghana where essential maternal and child health services, including vaccination, continue to be offered despite pressures imposed by the COVID-19 pandemic.

**Figure:**
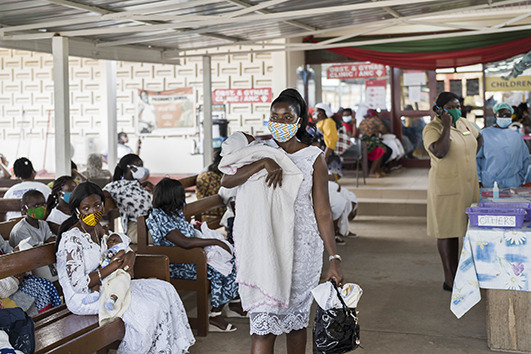
WHO/Blink Media - Nana Kofi Acquah

## Sepsis summary

WHO published its first global report on sepsis on 8 September. The report highlights the challenges faced in assessing the extent and impact of the condition. These include the fact that most published studies on sepsis have been conducted in hospitals in high-income countries, despite possibly 8 in every 10 sepsis cases occurring in LMICs. Other challenges include differing definitions of sepsis, varied diagnostic criteria and hospital discharge coding.

The report calls for greater efforts to sharpen the focus on sepsis through age-specific sepsis definitions, implementation of the ICD-11 classification of sepsis and promotion of high-quality epidemiological studies, especially in LMICs.

https://bit.ly/2RtaZmQ

## Cutting *trans* fats

Most countries are still not doing enough to remove industrially produced *trans*-fatty acids (TFA) from their food chains. According to a report published by WHO on 9 September, best-practice TFA policies that either virtually eliminate industrially produced TFA or ban partially hydrogenated oils are in effect in only 14 countries, representing 589 million people – 8% of the global population.

Consumption of TFAs are estimated to cause around 500 000 deaths per year due to coronary heart disease. WHO is working with Member States to replace TFAs with healthier oils and fats.

https://bit.ly/3iy2mmX

**Looking ahead**

October 24 - World Polio Day. https://bit.ly/2FG4Lxc

October 24–30 - International Lead Poisoning Prevention Week of Action. https://bit.ly/35FA46h

October 25–27 - World Health Summit. Berlin, Germany. https://bit.ly/33Np5W3

